# Small Noncoding RNAs in Reproduction and Infertility

**DOI:** 10.3390/biomedicines9121884

**Published:** 2021-12-12

**Authors:** Qifan Zhu, Jane Allyn Kirby, Chen Chu, Lan-Tao Gou

**Affiliations:** 1 State Key Laboratory of Molecular Biology, Shanghai Key Laboratory of Molecular Andrology, Shanghai Institute of Biochemistry and Cell Biology, Center for Excellence in Molecular Cell Science, Chinese Academy of Sciences, Shanghai 200031, China; zhuqifan@sibcb.ac.cn; 2 Department of Cancer Biology, Dana-Farber Cancer Institute, Boston, MA 02215, USA; Jane_Kirby@dfci.harvard.edu; 3 Department of Genetics, Blavatnik Institute, Harvard Medical School, Boston, MA 02115, USA

**Keywords:** small noncoding RNA, infertility, transgenerational epigenetic inheritance, biomarker, epididymosome, sperm

## Abstract

Infertility has been reported as one of the most common reproductive impairments, affecting nearly one in six couples worldwide. A large proportion of infertility cases are diagnosed as idiopathic, signifying a deficit in information surrounding the pathology of infertility and necessity of medical intervention such as assisted reproductive therapy. Small noncoding RNAs (sncRNAs) are well-established regulators of mammalian reproduction. Advanced technologies have revealed the dynamic expression and diverse functions of sncRNAs during mammalian germ cell development. Mounting evidence indicates sncRNAs in sperm, especially microRNAs (miRNAs) and transfer RNA (tRNA)-derived small RNAs (tsRNAs), are sensitive to environmental changes and mediate the inheritance of paternally acquired metabolic and mental traits. Here, we review the critical roles of sncRNAs in mammalian germ cell development. Furthermore, we highlight the functions of sperm-borne sncRNAs in epigenetic inheritance. We also discuss evidence supporting sncRNAs as promising biomarkers for fertility and embryo quality in addition to the present limitations of using sncRNAs for infertility diagnosis and treatment.

## 1. Introduction

Noncoding RNAs (ncRNAs) are transcripts that are not designed to be translated. Since their discovery in *C. elegans* [1,2], ncRNAs are identified as important regulators in various biological processes and disease states in animals. ncRNAs cover diverse RNA species that can be classified based on their functions, biogenesis, and size. ncRNAs with size smaller than200-nucleotides (nt) are classified as small noncoding RNAs (sncRNAs) that are comprised of housekeeping ncRNAs, including small nuclear RNAs (snRNAs), small nucleolar RNAs (snoRNAs) [3], as well as others with regulatory roles in gene expression, i.e., regulatory sncRNAs. Regulatory sncRNAs such as microRNAs (miRNAs), PIWI-interacting RNAs (piRNAs), and endogenous small interfering RNAs (endo-siRNAs), have been discovered for more than two decades. Their regulation and functions are further elucidated and better understood. Multiple platforms have been developed for the detection and measurement of sncRNAs, including real-time quantitative PCR (qPCR), microarray-based methods, and, most recently, high-throughput next generation sequencing (NGS) [4,5,6,7]. Novel categories of sncRNAs, such as transfer RNA (tRNA)-derived small RNAs (tsRNAs) (or tRNA-derived fragments, tRFs) [8,9] and ribosomal RNA (rRNA)-derived small RNAs (rsRNAs) (or ribosomal RNA-derived fragments, rRFs) [10], were identified with high-throughput small RNA sequencing. The mechanisms of their biogenesis and regulatory roles have only recently begun to emerge. Recent studies employing advanced technologies and bioinformatic analyses further expand the ncRNA field and shed light on the complexity of RNA modification, RNA structure, and dynamic interaction between RNA, DNA, and protein [11].

Gametes are the only cell type that transmit information across generations of sexually reproducing organisms. Studies have shown that miRNAs, endo-siRNAs, and piRNAs are critical regulators of germ cell development, as depletion of enzymes essential for their biogenesis leads to infertility [12,13]. sncRNAs regulate germ cell development with mechanisms at epigenetic, transcriptional, and post-transcriptional levels [14,15], performing distinct, sexually dimorphic functions in male versus female germlines. For example, piRNAs, although essential to male germ cell development, appear to be dispensable to murine oogenesis [16,17,18,19,20]. Further studies suggest that extracellular sncRNAs operate as mediators of somatic-germ cell communication and can be used as biomarkers for sperm and oocyte quality [21,22,23,24]. sncRNAs also play important roles during early embryonic development [25,26,27,28]. Increasing evidence indicates that sncRNAs, especially miRNAs and tsRNAs, may mediate the transmission of paternally acquired metabolic and mental disorders [29,30,31], presenting an important mechanism of disease predisposition in the developmental origins of health and disease (DOHaD) field.

Overall, sncRNAs demonstrate increasingly clear importance in mammalian reproduction. In this review, we discuss the regulation and significance of five classes of sncRNAs, miRNA, piRNA, endo-siRNA, tsRNA, and rsRNA, in the context of germ cell development and disease predisposition. We focus on these sncRNA subtypes as carriers of paternal epigenetic information across generations, as well as the discovery of novel sncRNA species such as tsRNA by NGS technologies. The potential utilization of sncRNAs as biomarkers for infertility diagnostics and the improvement of assisted reproduction will also be discussed in this review. Reproduction is a complex process involving not only germ cells but also gonadal somatic cells and multiple organs. The roles of sncRNAs in other reproductive diseases, such as reproductive cancer [32,33,34], ovarian and endocrinal disorders [35], and implantation failure [36,37], have been comprehensively reviewed and are not discussed in detail here.

## 2. Methods

We searched databases of PubMed, Scopus, and Web of Science for studies relevant to the role of sncRNAs in mammalian reproduction and infertility, with a focus on mammalian germ cell and epigenetic inheritance. The search was conducted before the end of September 2021. The following text words were used in the searching syntax: “sncRNA” or “small non-coding RNA” or “ncRNA” or “non-coding RNA” or “small RNA” or “miRNA” or “microRNA” or “piRNA” or “piwi-interacting RNA” or “endo-siRNA” or “tsRNA” or “tRNA-derived small RNA” or “tRF” or “tRNA-derived fragment” or “rsRNA” or “rRNA-derived small RNA” or “rRF” or “rRNA-derived fragment” or “sperm-borne RNA” or “extracellular RNA”, in combination with “mammalian” and “germ cell” or “primordial germ cell” or “PGC” or “oogenesis” or “oocyte” or “spermatogenesis” or “spermiogenesis” or “spermatogonia” or “spermatocyte” or “spermatid” or “epididymis” or “epididymosome” or “extracellular vesicles” or “early embryo” or “zygote” or “embryo culture” or “inheritance”. For example, the searching strategy in Scopus was listed as the following: TITLE-ABS-KEY (“sncRNA” OR “small non-coding RNA” OR “ncRNA” OR “non-coding RNA” OR “small RNA” OR “miRNA” OR “microRNA” OR “piRNA” OR “piwi-interacting RNA” OR “endo-siRNA” OR “tsRNA” OR “tRNA-derived small RNA” OR “tRF” OR “tRNA-derived fragment” OR “rsRNA” OR “rRNA-derived small RNA” OR “rRF” OR “rRNA-derived fragment” OR “sperm-borne RNA” OR “extracellular RNA”) AND ALL (“mammalian”) AND TITLE-ABS-KEY (“germ cell” OR “primordial germ cell” OR “PGC” OR “oogenesis” OR “oocyte” OR “spermatogenesis” OR “spermiogenesis” OR “spermatogonia” OR “spermatocyte” OR “spermatid” OR “epididymis” OR “epididymosome” OR “extracellular vesicles” OR “early embryo” OR “zygote” OR “embryo culture” OR “inheritance”). All studies associated with the discovery, regulation, and function of sncRNAs in mammalian germ cell and epigenetic inheritance were then manually selected based on their publication year and the number of their citing articles, as we aim to include the most recent studies with high impact and novelty. The references and cited articles of included studies were also manually searched. The quality of these studies was assessed manually as the included studies, depending on their hypotheses and purposes, varied largely in methods and analyses.

## 3. Biogenesis and Functions of sncRNAs

miRNAs, endo-siRNAs, and piRNAs are the most well-studied classes of sncRNAs. They are mainly distinguished by their biogenesis mechanisms and interacting partners. In animals, the canonical pathway of miRNA processing begins with the transcription of primary miRNA (pri-miRNA) by RNA polymerase II. Processed by the Drosha-DGCR8 complex, the hairpin-shaped pri-miRNAs are trimmed to ~65 nt precursor miRNAs (pre-miRNAs) in the nucleus [38]. Transported into the cytoplasm by Exportin 5-Ran GTPase, pre-miRNAs are further cleaved by DICER into ~22 nt miRNA duplexes, which are loaded onto AGO proteins by DICER, TRBP, and/or PACT [39]. The mature (or major) miRNA remains on AGO proteins, forming the miRNA-induced silencing complex (miRISC). Within miRISC, miRNA functions as the guide molecule targeting the 3′ untranslated region (3′ UTR) of mRNAs via imperfect base pairing, leading to mRNA decay or repression of translation. The other strand (minor) is mostly degraded [40]. Individual miRNAs can generate isoforms (isomiRs) that vary in length and/or sequence. Consequently, these isomiRs may differ from each other in structural stability, how they are loaded into the miRISC, and their interaction with targets [28,41,42]. A recent study further suggests that miRNA may also regulate the RNA modification of mRNA transcripts. It was reported that miRNAs modulate the binding of N^6^-methyladenosine (m^6^a) writer, METTL3, to mRNA via a base-pairing mechanism and, in turn, regulate m^6^a abundance [43].

Endo-siRNAs, which are slightly shorter than miRNAs (~21 nt), were ubiquitously found in higher eukaryotic models [44,45,46]. The generation of endo-siRNA precursors is less conserved among species; in worms and plants this process is frequently dependent on RNA-dependent RNA polymerases (RdRPs) [47,48,49], while RdRPs are in general not required for endo-siRNA generation in flies and mammals. Distinct from exogenous siRNAs (exo-siRNAs) arising from ectopically introduced transcripts such as viral RNA, endo-siRNA precursors are often sense–antisense transcript pairs or long stem-loop structures that originated from repetitive elements and pseudogenes [13,44,45]. Endo-siRNAs also demonstrate differences from miRNAs as endo-siRNA precursors are long double-strand RNAs (dsRNAs) and their processing is independent of Drosha and DGCR8 [50,51]. Endo-siRNA precursors are cleaved by DICER in cytoplasm. Similar to miRNA, endo-siRNA are loaded into AGO-family protein such as AGO2, forming the RISC complex that regulates target transcripts by inducing RNA cleavage [13] and also translational repression [52].

piRNAs, as their name indicates, are associated with PIWI proteins, a germline-specific and highly conserved group of the AGO family. These small RNAs are around 24–32 nt in length. The 5’ end of piRNAs maintain a strong preference for uridine [53,54] while the 3’ end often show 2′-O-methylation mediated by Hen1 (Henmt1 in mice) [55,56,57]. Biogenesis of piRNAs is most commonly investigated in flies and mice. Distinct from miRNAs and endo-siRNAs, the generation of piRNAs from single-stranded precursors does not depend on DICER and Drosha. These single-stranded precursors are produced by RNA polymerase II from repetitive elements and loci called piRNA clusters, which are then exported into cytoplasm via interaction with UAP56 and Vasa [58]. The processing of piRNA precursors involves two mechanisms, i.e., the primary processing and the ping-pong cycle that post-transcriptionally amplifies piRNAs [59,60]. In brief, during the primary processing, long single-stranded precursors are converted into small RNAs with a clear preference for 5′ uridine and 2′-O-methylation at the 3′ end. Activity of several nucleases, as well as their targeting and recruiting mechanisms, are key to this process. This includes the endonuclease Zucchini (also known as MitoPLD or PLD6) [61,62,63,64] and TDRKH-mediated recruitment of 3′ exonuclease Trimmer (PNLDC1 in mice) for trimming [65,66,67]. TDRKH is a Tudor-domain containing (TDRD) protein. Multiple members of the TDRD protein family show interaction with PIWI proteins and are essential for piRNA biogenesis and retrotransposon repression [68,69]. Functional piRNAs generated via primary processing form complexes with PIWI proteins and initiate the ping-pong cycle, during which the endonuclease activity of PIWI proteins is crucial [70,71]. The most documented role of piRNAs is to repress transposable elements (TE) in both post-transcriptional and epigenetic ways [72]. Multiple studies reveal that piRNAs, by associating with PIWI proteins, also form a piRNA-induced silencing complex (piRISC) and mediate mRNA degradation similar to miRNA and endo-siRNA [73,74,75]. Recent studies further indicate a translation-activating role of piRNA/PIWI in mouse spermiogenesis [15] and Drosophila embryogenesis [76].

A portion of miRNA and piRNA subtypes are suggested to be derived from tRNA- and/or rRNA-encoding regions, supporting the concept that tsRNAs and rsRNAs are functional sncRNAs [77,78,79,80]. Evidence surrounding the biogenesis and functions of tsRNAs and rsRNAs has been growing rapidly in the past decade, yet far less is known about them compared to the three aforementioned sncRNA species. Mature tRNAs or tRNA precursors can be cleaved at different sites to generate tsRNAs. The preferred sites and cleavage events are determined by the structure of tRNA and regulated by RNA modifications and RNases, including DICER and Angiogenin (ANG) [81,82,83]. For example, DNMT2 and NSUN2-mediated 5-methylcytosine (m^5^C) modification of tRNAs increases the stability of some tRNAs, making them less likely to be cleaved into tsRNAs [84,85]. TET2, on the other hand, promotes the conversion of m^5^C on tRNA to 5-hydroxymethylcytosine (hm^5^C) on tRNA, displaying an opposite effect to NSUN2 on regulating the tsRNA profile [86]. As a result of different cleavage sites, tsRNAs can be mapped to different regions of tRNAs and are classified into several types, including tRNA halves (also called tiRNAs or tiRs) (31–40 nt) and smaller (14–30 nt) 5′ tRF, 3′ tRF, tRF-1, tRF-2, and internal tRFs (i-tRFs) [8,11,87,88]. rsRNAs, which are derived from both nucleus- and mitochondria-encoded rRNAs, have been mostly overlooked during small RNA analysis. Recent studies indicate the biogenesis of rsRNAs are non-random and regimented, as different types of rRNAs exhibit preferred cleavage sites and generate rsRNAs with favored lengths [89]. rsRNA profiles also appear to be highly context specific [90,91]. The regulation of rsRNAs, although largely undiscovered, may show some similarity with tsRNAs, as both tRNA and rRNA are ancient RNA classes with highly conserved secondary structures, sensitive to environmental changes. RNases, such as yeast Rny1p and mammalian SLFN13, are suggested to have nuclease activity towards both tRNA and rRNA [92,93]. As with the sncRNAs mentioned above, tsRNAs and rsRNAs may also be associated with AGO proteins, mediating post-transcriptional regulation for retrotransposons and protein-coding genes [10,90,94]. tsRNAs and rsRNAs may also function using more diverse mechanisms, including targeting, mimicking, or replacing tRNAs and rRNAs, respectively, based on the sequence and structure similarity [11,95,96,97].

## 4. Functions of sncRNAs during Mammalian Germ Cell Development

During spermatogenesis in mouse testes, piRNA is the dominant species of the small RNA population. piRNA-related factors often locate to a germline-specific, electron-dense structure known as nuage [61]. Mutations of piRNA pathway components including PIWI proteins lead to male sterility, indicating that piRNAs are essential for male fertility [18,19,61,70]. Based on the stages they appear and their functions, piRNAs detected during mice spermatogenesis can be divided into three sets: the first set of piRNAs (26–28 nt) emerges in fetal testis and initiates transposon silencing via de novo DNA methylation [71,98]; the second set, pre-pachytene piRNAs (26–27 nt), derives mainly from the 3′UTR of mRNA in neonatal testis, of which their functions remain unknown [99,100,101]; and the third set, pachytene piRNAs (26–30 nt), accumulating from the pachytene phase of meiosis, is highly diverse and abundant. The pachytene piRNAs are generated from ~100 well-defined autosomal clusters that are relatively depleted of transposon sequences [100] and are marked by a first exon with unusual length (≥10 kb) [102]. Transcription factor A-MYB (also known as MYBL1) is suggested to initiate the transcription of pachytene piRNA clusters [100,103]. Transcriptional elongation factor BTBD18 is also required for the generation of piRNAs from ~50 of those clusters [104]. Mutation of those two factors also results in male infertility [100,104]. Recent studies have proposed several roles of pachytene piRNAs including (1) regulating meiotic progression by cleaving meiotic mRNA such as *TDRD1* [74,105]; (2) eliminating bulk mRNAs in spermatids via miRNA-like mechanism [73]; and (3) activating translation of a subset of spermiogenic mRNAs, which contain AU-rich elements in the 3′ UTRs, via imperfect base pairing in spermatids. This piRNA-activating translation of mRNAs, such as *Agfg1* and *Tbpl1*, is implied to be critical for acrosome formation during spermiogenesis [15]. Later studies generated mice with disruption in the promoter of specific pachytene piRNA clusters, revealing detailed roles of pachytene piRNAs in regulating spermiogenesis. Deletion of the promoter of a conserved pachytene piRNA locus on Chr6 (termed *pi6*) induces defective sperm acrosome function in male mice, manifesting as impaired capacitation and defects in egg fertilization. These sub-fertile male mice also produce embryos with reduced viability in utero [106]. Similarly, male mice lacking another pachytene piRNA cluster on Chr18, which is one of the clusters targeted by BTBD18, are sterile due to sperm acrosome overgrowth. Acrosome overgrowth is likely to be related to the increased abundance of GOLGA2 transcript, leading to severe sperm head deformation and failure in egg fertilization [107].

Unlike in *Drosophila* and zebrafish where the piRNA pathway is essential for both spermatogenesis and oogenesis [108,109], mutation of key components of the piRNA machinery, such as MIWI (PIWIL1) and MILI (PIWIL2), in mouse oocyte development does not affect female fertility. However, almost complete loss of piRNAs, which is observed in *Mili* mutant females, does lead to derepression of some retrotransposons in mouse oocytes, suggesting an uncoupling between retrotransposon expression and fertility [16,20,110]. Another key PIWI protein, PIWIL4 (MIWI2 in mice and HIWI2 in humans), which is essential for spermatogenesis [19], is negligibly expressed in mammalian oocytes [110,111]. Notably, mice and rats do not encode a fourth PIWI protein, PIWIL3 (HIWI3 in humans), which is oocyte specific and detected during oogenesis of many other mammalian species [111,112]. A recent small RNA sequencing of single oocytes revealed a specific class of sncRNAs (~20 nt) associated with PIWIL3 during human and monkey oogenesis. This particular sncRNA class, termed oocyte short piRNAs (os-piRNAs), exhibits feature similar to both piRNAs and endo-siRNAs [113]. Similarly, in hamsters, a class of short piRNAs (~19 nt) are found to be uniquely associated with phosphorylated PIWIL3 in MII oocytes [111]. Although both studies suggest that the PIWIL3-associated piRNAs may target TEs, the function of PIWIL3 and its associated piRNAs in oogenesis remain largely unexplored [111,113].

miRNAs are also necessary for mammalian primordial germ cell (PGC) development and different stages of spermatogenesis. Depletion of miRNA processing components (i.e., DICER1, DROSHA, or DGCR8) in mouse germ cells leads to massive loss of miRNAs and male infertility [12,114,115,116,117,118]. Various miRNAs have been predicted in silico and/or experimentally proven to target protein-coding genes that are important for gametogenesis. A single miRNA may have multiple targets and, vice versa, a singular mRNA may be targeted by several miRNAs. This complex miRNA–mRNA regulatory network is stage- and cell type-specific. Aberrant expression of miRNAs at each stage of spermatogenesis leads to distinctive defects [119]. For example, during the differentiation of PGCs to spermatogonia cells, several miRNAs potentially target and regulate the abundance of *Pten* transcripts which are suppressors of proliferation [77]. During male meiosis in spermatocytes, miRNAs are also extensively involved in events such as meiotic sex chromosome inactivation (MSCI) and apoptosis [120,121]. miRNAs in mature spermatozoa also show association with male fertility [122]. Although general biogenesis of miRNAs is well-understood, fine-tuning of specific miRNAs in a spatiotemporal manner remains relatively unexplored. RNA modification, in specific m^6^a, has been proposed to regulate the recognition and processing of pri-miRNA [123,124]. Mutation of multiple m^6^a writers and readers results in spermatogenic defects [125,126,127,128]. Intriguingly, increased level of m^6^a in human mature spermatozoa may be indicative of spermatogenic impairments [27]. Still, whether m^6^a modification occurs on miRNAs and other sncRNAs during spermatogenesis is largely unknown [125,126,127,128]. Another possible regulator of miRNAs, circular RNA (circRNA), is detected in human testis, spermatozoa, and seminal plasma [129,130] as well as mouse spermatogenic cells [131]. circRNAs are widely recognized as miRNA sponges, i.e., competing transcripts that sequester and inhibit miRNA activity. A testis-specific circRNA derived from male sex-determining gene *Sry* was shown to contain 16 sites for miR-138, acting as its sponge [132]. Furthermore, circRNAs were shown to respond to toxicity and stress in the germline and may play protective roles in germline development by sponging miRNAs [133,134,135].

While miRNAs are required for both male and female PGC development [12,118] and detectable throughout oogenesis, their functions in developing oocytes are predominantly suppressed. DICER1 and AGO2 are required for both miRNA and endo-siRNA pathways, and depletion of either in mouse oocytes leads to meiotic spindle defects. However, *Dgcr8* mutant female mice, which display defects in the canonical miRNA pathway while retaining a fully functional endo-siRNA pathway, exhibit no meiotic defect and are fertile. Although DGCR8-depleted oocytes show profound loss of miRNAs such as DICER1-depleted oocytes, they are relatively similar to wild-type (WT) oocytes at transcriptomic level [136,137]. On the other hand, a decrease in endo-siRNAs, observed in *Dicer1* and *Ago2* mutant but not *Dgcr8* mutant oocytes, increases levels of both transposon and protein-coding transcripts that are complementary to those endo-siRNAs [13]. These observations suggest that endo-siRNAs, rather than miRNAs, are critical for female meiosis. The optimal biogenesis of endo-siRNA in mouse and rat oocytes is further ensured by an N-terminally truncated isoform of DICER (DICER^O^), with higher cleavage activity compared to somatic DICER (DICER^s^). Expression of this mouse- and rat-specific DICER^O^ is driven by an oocyte-specific promoter derived from retrotransposition [138]. Using a low-input sequencing technique (LACE-seq) that identifies RNA networks associated with specific proteins, a recent study reveals the direct targets of AGO2 in mouse MII oocytes. Surprisingly, many AGO2 targets are unaltered at the transcriptional level in AGO2- or DICER^O^ -deficient oocytes, suggesting endo-siRNAs and AGO2 may predominantly mediate translational regulation. Further proteomics analysis confirmed this translation regulatory function of the endo-siRNA-AGO2 complex in mouse oocytes [52].

Numerous endo-siRNAs are also detected in mouse spermatogenic cells [50]. Using a similar strategy, i.e., comparing the phenotypes of DICER1- or DGCR8-depleted male germlines, Zimmermann et al. suggest that endo-siRNAs have some effect on mouse spermatogenesis. However, endo-siRNAs are insufficient for male meiosis, as *Dgcr8* mutant male mice with a defected miRNA pathway but an intact endo-siRNA pathway are infertile, albeit with slightly less severe phenotypic outcome as compared to *Dicer1* mutant mice [115]. dsRNAs, which are processed by DICER into endo-siRNA, are particularly abundant in pachytene spermatocytes. The expression profile of dsRNAs in testis shows a significant association with endo-siRNAs and antisense transcripts and is, in general, greatly altered from those in somatic tissues [139]. Over 80% of endo-siRNAs in mouse MII oocytes were mapped to repetitive elements [140], whereas the majority of the dsRNAs in testis were potentially derived from protein-coding regions, implying a potentially different endo-siRNA pathway between males and females. Nevertheless, the function of endo-siRNAs in spermatogenesis remains largely unexplored [139].

**Figure 1:**
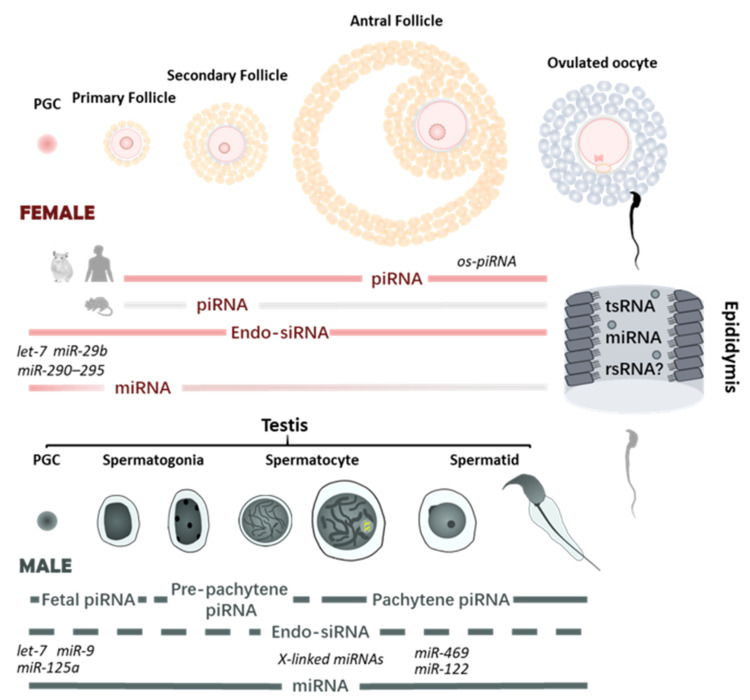
sncRNA profiles during mammalian gametogenesis. Spatiotemporal profiles of sncRNA pathways have been investigated during mammalian germ cell development. In male, piRNAs and miRNAs are essential to spermatogenesis and spermiogenesis. Endo-siRNA pathway is observed during spermatogenesis, yet its function remains largely unknown (shown as dashed line). Spermatozoa also gain sncRNAs during their transit through the epididymis. In female, only endo-siRNAs and potentially noncanonical miRNAs are crucial for mouse oogenesis. As knockout of key components of piRNA and canonical miRNA pathways does not affect female fertility, the activities of those pathways are suggested to be suppressed during mouse oogenesis (shown as grey lines). However, recent studies suggest that piRNA pathway is critical for female fertility in golden hamster and potentially primates, with PIWIL3-interacting oocyte short piRNAs (os-piRNAs) detected in those non-rodent mammals, raising the possibility that piRNAs and PIWI genes may also be required for human fertility in women.

Taken together, recent studies employing advanced technologies have revealed the dynamics and complexities of sncRNA pathways during mammalian germline development and further emphasized their extensive influence on reproduction (Figure 1). Different sncRNAs seem to carry out unequal contributions to germline development between genders and among species. piRNAs, miRNAs, and, to a lesser extent, endo-siRNAs contribute to spermatogenesis, while only endo-siRNAs and possibly noncanonical miRNAs are required for mouse oogenesis. Comprehensive knowledge of the sncRNA network during spermatogenesis and oogenesis may provide new clues for the pathogenesis and potential treatment of idiopathic infertility, which is proposed to be the most common diagnosis of infertility [141,142,143].

## 5. Contribution of Epididymosome-Encapsulated sncRNAs to Sperm-Borne sncRNAs

As the sperm leaves the testis and transits through the epididymis, the process of sperm maturation occurs, during which spermatozoa acquire full fertility capacity. The epididymis is a long, highly coiled tubule linking the testis and vas deferens. Conventionally, mammalian epididymides are comprised of three main regions (caput, corpus, and cauda), and each region can be subdivided into a number of morphologically and transcriptionally distinct segments [144,145]. Epididymosomes, the extracellular vesicles (EV) secreted by epididymal epithelial cells, are composed of lipids, proteins, and ncRNAs. Epididymosomes contribute to the sperm maturation process by mediating the crosstalk between the epididymis and the passing spermatozoa, including the transfer of the environmental information from epididymal epithelial cells to sperm [146,147,148,149]. This soma-germline crosstalk provides an additional mechanism affecting male fertility and embryo development [21,150,151]. The content of epididymosomes, including ncRNAs, exhibits segment to segment variations [21,152]. Although fusion of epididymosomes with spermatozoa has been observed in vitro [21], the underlying mechanisms regulating such epididymosome-sperm crosstalk remain largely unknown. As sperm transits from the testis to epididymis, tsRNAs become the dominant sncRNA (~80%) present in mouse sperm [21]. Similarly, ~56% of sncRNAs in mature human spermatozoa are annotated to tsRNAs, of which ~75% are 5′-tRNA halves, i.e., tsRNAs derived from the 5′half of mature tRNAs [23]. In addition, both human and mouse mature spermatozoa contain abundant rsRNAs, which were initially overlooked and filtered out during analysis [153]. The majority of these rsRNAs in mouse and human spermatozoa are derived from 28S rRNA [23,153]. As spermatozoa are generally believed to be transcriptionally silent when leaving the testis, the remodeling of sncRNA repertoire in maturing sperm may be causally linked to the uptake of epididymosome-encapsulated tsRNAs and potentially rsRNAs. Maturing sperm also absorb miRNAs that are shown to be abundant in the cauda epididymosome [21,154]. Notably, a substantial increase in piRNAs is detected in cauda spermatozoa independent of the epididymosome-sperm trafficking, as piRNAs are generally scarce in epididymosomes [152]. Overall, this complex sncRNA profile of mature spermatozoa and the information it carries is a major part of the “sperm RNA code” [155].

Although regulatory mechanisms of tsRNA and rsRNA in testicular sperm or epididymis remain largely unknown, several proteins may be involved in tsRNA biogenesis, including RNA modifiers DNMT2 and NSUN2 [84,85]. Given that RNA modifications are rich in tRNAs and rRNAs and contribute to the generation of tsRNAs and rsRNAs [84,85], the resulting tsRNAs and rsRNAs are likely to inherit those modifications. Indeed, multiple RNA modifications such as m^5^C and N^2^-methylguanosine (m^2^G) have been detected in sperm tsRNAs [29,84]. A recent study further reports previously undetected sncRNAs in multiple tissues when sequencing-aborting RNA modifications are removed before small RNA sequencing (PANDORA-seq). Most of the newly detected sncRNAs are tsRNAs and rsRNAs [156]. RNA modifications have been known to affect RNA stability and functions and therefore may expand the complexity of the sperm RNA code. Apart from the regulation of sncRNA composition and associated RNA modification, the compartmentalization of sperm sncRNAs may also be relevant to sperm RNA code as it is correlated with the origins and functions of sncRNAs. A greater proportion of rsRNAs is observed in mouse mature sperm compared to mature sperm head, suggesting that a portion of rsRNAs may be associated with the sperm tail which is rich in mitochondria. Interestingly, the rsRNA-generating positions on mitochondria-encoded 12S and 16S rRNA also appear to be different between the mature sperm and mature sperm head [156]. In addition, a relatively greater amount of piRNAs are detected in the sperm tail than in the sperm head [21]. It is tempting to speculate that mitochondria in the sperm tail may retain some transcriptional activities and therefore provide an additional source of sncRNAs in mature sperm [80].

## 6. Sperm-Borne sncRNAs and Their Potential Roles in Developmental Origins of Health and Disease (DOHaD)

**Figure 2:**
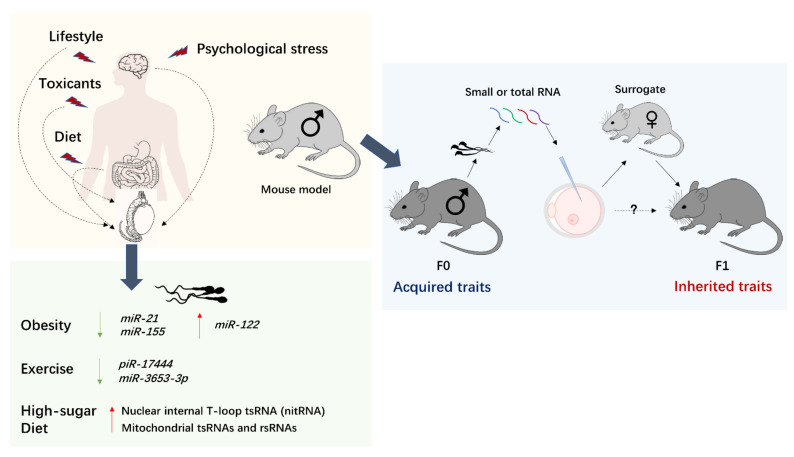
Environmental factors, including psychological trauma, diet, lifestyle choices, and exposure to toxicants, not only affect the exposed individuals but also elicit multigenerational effects. These environmental exposures induce alterations in the sperm sncRNA profile of humans. The roles of sperm sncRNA in transmitting acquired traits are mainly investigated in mouse models. Phenotypic changes are induced in those mouse models, mimicking various environmental exposures in humans. Subsequently, total RNA or the small RNA fraction extracted from mature spermatozoa of the phenotypically-changed mouse (F0) is injected into normal zygotes (i.e., generated by unexposed parents). Those RNA-injected zygotes are implanted in unexposed surrogates for further development. The resulting pups or adult mice (F1) are evaluated for phenotypes.

Immediately following fertilization, the early embryo remains transcriptionally quiescent. Transcriptional activity is first detected in mid to late one-cell mouse zygote and at 4- to 8-cell stage in human zygote. This resumption of transcription from the zygotic genome is termed maternal to zygotic transition (MZT). Embryonic development before MZT is therefore dependent on maternally stored transcripts and sperm-borne RNAs. In recent years, increasing evidence in mouse and rat models has established the existence of epigenetic inheritance in mammals, where the impact of parental exposure to environmental changes is transmitted to the offspring via epigenetic information in the germline [157]. Sperm-borne sncRNAs, which are sensitive to environmental stress, are proven to be one of the carriers of paternally acquired characteristics and impact the health of future progeny (Figure 2). These environmental stresses primarily include metabolic stress, psychological trauma, and exposure to toxicants [157,158,159]. These features of sperm-borne sncRNAs show potential interrelatedness with the DOHaD concept, a theory focusing on the effects of environmental exposures during early development and the susceptibility (or developmental origins) of non-communicable diseases during adulthood, such as type 2 diabetes and depression [160,161] (Table 1). Although mechanistic evidence remains unelucidated, research on the epigenetic inheritance of acquired traits and the roles of sperm-borne RNA may contribute to our understanding of the epidemiology of obesity and type 2 diabetes, which currently cannot be fully explained by genetic factors [162,163,164].

### 6.1. Sperm-Borne sncRNAs Are Carried into Oocytes and Contribute to Early Development

In general, evidence in multiple species and with various methods indicates that sperm-borne sncRNAs are most likely to be carried into oocytes during fertilization and can impact early development. miRNA- and endo-siRNA-deficient spermatozoa generated by germline-specific *Dicer1* and *Drosha* conditional knockout (cKO) mice can fertilize WT eggs but display moderate reduction in developmental potential from the pronuclei stage and abnormal gene expression during preimplantation development. This diminished developmental potential is enhanced by injecting WT sperm-borne small RNAs or total RNA, suggesting that paternal miRNAs and endo-siRNAs may contribute to the maternal transcript degradation and MZT [190]. Using a highly sensitive sequencing method, five miRNAs that were prominently expressed in sperm were found to be upregulated in the one-cell zygote compared to parthenogenetic one cell, suggesting those miRNAs are carried into oocytes by sperm during fertilization [140]. In bovine, injecting sperm-borne sncRNAs in somatic cell nuclear transfer (SCNT) embryos showed decreased H3K9me3 level and increased acetylation of α-tubulin K40, which subsequently lead to postponedfirst embryo cleavage and enhance developmental competence of SCNT embryos [191]. Further experiments indicate that a sperm-enriched miRNA in bovine, miR-202, may be involved in regulating the timing of the first cleavage by targeting SEPT7, a cytoskeletal GTPase required for mitosis [192]. In porcine, depletion of a specific functional tsRNA group derived from tRNA^Gln-TTG^ (termed Gln-TTG) in IVF oocytes by injecting the antisense strand led to aberrant first cleavage. The depletion of Gln-TTGs in porcine IVF oocytes also resulted in downregulation of a LINE1 family retrotransposon, L1M3b [193].

It is worth noting that “readouts” of the impact of sperm-borne sncRNAs on early development, such as fertilization rate and blastocyst rate, are largely affected by the type of experimental procedures used in studies, including the intracytoplasmic sperm injection (ICSI) procedure and methods used to “neutralize” sperm-borne sncRNAs. Consequently, discrepancies have been reported between studies. For example, the sperm-borne miRNA miR-34c-5p was initially reported as essential for the first cleavage in mouse embryos [194] but was later determined to be dispensable during early development in another study [195]. Liu et al. used antisense strands for miRNA depletion, while Yuan et al. used a miRNA knockout (KO) model. It was suggested that the antisense strands, if overdosed, may have off-target effects where developmental arrest is observable. Therefore, precise targeting of sncRNAs of interest is crucial to avoid technological bias and to appropriately interpret the resulting phenotype [195]. Another discrepancy concerns the necessity of sncRNAs from cauda epididymosome for early development [21,196,197,198,199]. Conine et al. initially showed that embryos generated via ICSI with sperm extracted from caput epididymis had abnormal expression and were arrested shortly after implantation. This embryo lethality can be rescued by microinjection of small RNAs from cauda epididymosome, suggesting that those small RNAs can be transferred into an embryo by sperm and maintain essential roles in embryonic development [196]. However, studies from different groups report that both caput and cauda sperm can generate full-term pups and embryo lethality observed by Conine et al. is mainly due to the “needle shearing” procedure used in their study to prepare the sperm head for ICSI. Therefore, the acquisition of sncRNAs from cauda epididymosomes by sperm is not necessary for their competence to fertilize and support embryonic development [197,199]. Still, it cannot be ruled out that small RNAs from cauda epididymosomes may have a functional role in embryonic development, given that they enhance embryo survival rates in the specific condition that Connie et al. used [198].

The above studies focus on the existence and functional mechanisms of sperm-borne sncRNAs in early embryos. In the meantime, other studies begin to investigate sperm-borne sncRNAs with a slightly different perspective, i.e., whether they can act as carriers of paternally acquired information that leads to phenotypic changes in offspring. The first pieces of evidence that outline the role of sncRNAs as hereditary epigenetic modifiers are presented in paramutation mouse models, where injection of miRNAs, which may not be sperm-borne naturally, into fertilized eggs was used as a signal to induce the hereditary transfer of the paramutated state [200,201,202]. There is a growing body of evidence in support of sperm-borne sncRNAs exhibiting sensitivity to various environmental factors. Abnormal changes in the sperm-borne sncRNA profile due to environmental stress were shown to mediate the inheritance of paternally acquired adverse phenotypes in mammals. In several studies, this inheritance of acquired phenotypes appears to be transgenerational. However, mechanistic evidence, i.e., detailed roles of sperm-borne sncRNAs, has mostly been investigated in an intergenerational manner.

### 6.2. Sperm-Borne sncRNAs Mediate Transmission of Diet-Induced Phenotypes into Offspring

Since 2015, a growing number of studies have demonstrated inheritance patterns of diet-induced metabolic disorders via sperm-borne sncRNAs. Grandjean et al. injected testis and sperm RNA of mice kept on a Western-like diet (WD, i.e., high fat and high sugar) into a normal zygote, both of which resulted in progenies developing WD-induced metabolic phenotype. Injection of a high amount of one specific miRNA that is increased in the testis of WD-fed mice into normal zygote similarly induced metabolic abnormalities in the offspring which were subsequently transmitted to the next generation [172]. Strikingly, a more recent study reported that when male mice were fed with WD for five consecutive generations, the successive four generations of male descendants, when fed with normal diet, can develop a ‘healthy’ overweight phenotype, i.e., overweight but with normal glucose metabolism and without fatty liver. The sperm sncRNA signatures observed in the first generation of WD-fed mice, including an increased proportion of rsRNAs, tend to disappear in the fifth generation of WD-fed mice. These results suggest that a coping mechanism in response to metabolic stress can evolve within five generations [173].

Chen et al. observed that in sperm of male mice kept on a high-fat diet (HFD), a subset of 30–40 nt 5′-tRNA halves exhibit changed expression profiles as well as RNA modifications (m^5^C and m^2^G). When injected with 30–40 nt small RNAs from the sperm of HFD-mice, normal zygotes develop into adults with HFD-induced metabolic disorders. Moreover, RNA modifications may also be informative as injection of synthetic small RNAs did not reproduce the phenotypes in offspring [29]. A recent study suggests that some of those 30–40 nt small RNAs are rsRNAs, in addition to tsRNAs [156]. The same group reported that DNMT2-mediated modification is required for the generation of tsRNAs and rsRNAs in spermatozoa, thus emphasizing their importance in the transmission of diet-induced metabolic disorders. However, progenies of the Dnmt2 KO parents with the fathers fed on HFD still display some metabolic defects including insulin resistance, calling attention to the existence of an uncharted intergenerational mechanism [84]. HFD has also been used to investigate the effect of maternal overnutrition throughout preconception, gestation, and lactation. This maternal HFD is shown to affect three generations of offspring via the paternal lineage. Intriguingly, the maternal HFD induces both metabolic and mental phenotypes, with clear sexual dimorphism observed in the third generation. The third generational males with maternal HFD ancestors exhibit metabolic defects including obesity and insulin resistance, while the females exhibit addictive-like behaviors [175]. Microinjection of sperm total or tsRNAs from F1 with maternal HFD ancestors into normal zygotes generate offspring with both metabolic and mental phenotypes, although the phenotypes present in the tsRNA-injected group are subtler compared to those of the total RNA-injected group. This finding indicates potential contributions by other ncRNAs, such as long noncoding RNAs (lncRNAs). Surprisingly, some sperm tsRNAs are predicted to target genes involved in addiction pathology, yet whether and how they contribute to the observed phenotypes is unknown [174].

Sharma et al. used a mouse model consuming low-protein diet (LPD), which they report induced altered liver gene expression in F1 offspring [203]. Altered sncRNA profile was also observed in the sperm of LPD-mice, presenting decreased levels of miRNA let-7 and increased 5′ tRFs from glycine tRNAs (28–34 nt). A specific type of 5′ tRF derived from tRNA^Gly-GCC^ was found to supress genes associated with MERVL, an endogenous retroelement. Those 5′ tRFs were supposedly derived from the epididymis rather than the testis, as tRFs were primarily detected in the epididymis [21,30]. A recent study further indicated that LPD led to an increased level of reactive oxygen species (ROS) in testicular germ cells (TGCs) which indirectly enhanced expression of some tRNAs in TGCs. However, higher level of tRNA in testis does not appear to be correlated with the increased level of tsRNA in spermatozoa of LPD mice, alluding to involvement of additional sources such as epididymosomes. Whether and how the increased level of tRNAs in TGCs contributes to the sncRNA profile in spermatozoa and if increased ROS levels also occur and contribute to tRNA cleavage in the epididymis remains to be explored [171,204].

Similar to the aforementioned observations in mice, the sperm sncRNA profile in humans is sensitive to changes in metabolic health [177] as well as environmental changes, including diet intervention. In one study, participants were introduced to one week of a healthy diet followed by one week of a high-sugar diet (HSD). Upon conclusion of this study, Nätt et al. observed that mitochondrial-derived sperm tsRNAs and rsRNAs were upregulated significantly after the one-week of HSD. A specific type of tsRNA derived from the internal T-loop was also sensitively elevated after HSD. Interestingly, sperm motility was generally enhanced after the two-week diet intervention, gesturing towards a novel and intriguing theory regarding shared regulatory factors between sperm motility and sncRNAs [180,205]. A most recent study investigated the effects of a 6-week diet intervention, which was enriched in vitamin D and omega-3 fatty acids, on the sncRNA profile of human spermatozoa. Expression of a few sncRNAs, especially piRNAs, was altered post-intervention. Some of those altered sncRNAs are predicted to target genes involved in fatty acid metabolism and vitamin D response [179].

### 6.3. Sperm-Borne sncRNAs Mediate Transmission of Psychological Condition-Induced Phenotypes into Offspring

Psychological condition, including early traumatic experience, stress, and depression, is another transgenerationally influential factor that has been extensively linked to alteration in sperm sncRNA profiles. Using mice experiencing unpredictable maternal separation combined with unpredictable maternal stress (MSUS), Gapp et al. have investigated the transgenerational effects of early trauma. Abnormal behavioral and metabolic traits induced by early traumatic experience, such as depressive-like behavior and insulin hypersensitivity, can be transmitted to the third generation. Microinjection of total RNA from sperm of MSUS-exposed mouse (F1 MSUS) into normal zygote generated offspring that recapitulate these traits. Several miRNAs have been shown to be upregulated in F1 MSUS sperm, while some piRNAs were downregulated. However, the miRNA signatures observed in F1 MSUS sperm disappeared in F2 and F3, suggesting that additional mechanisms are involved to maintain and transmit the traits after F2 [167]. Interestingly, the following study has indicated that the profiles of long RNAs, including mRNA and lncRNA, are also altered in F1 MSUS sperm. Microinjection of long RNA (>200 nt) and small RNA (<200 nt) fractions from F1 MSUS sperm reproduce different traits, with long RNA fraction reproducing traits related to food intake, risk-taking, and glucose response to insulin while sRNA fraction leading to increase in body weight and increased trends of behavioral despair [168].

In a mouse model of paternal chronic stress prior to conception, the offspring often exhibited a reduced hypothalamic–pituitary–adrenal (HPA) stress axis response and transcriptional changes in the hypothalamus cells [165]. Expression of nine miRNAs increased in the sperm of chronic stress-exposed mice. Remarkably, microinjection of this set of nine miRNAs reproduced the blunted HPA stress response in adult offspring, which was not observed in a single-miRNA injection with the same total concentration. These findings suggest a specific miRNA network may be required for transmission of stress-induced phenotypes [31]. A second mouse model mimics paternal stress via intake of additional corticosterone (CORT), the stress hormone. The male founder mice were administered water supplemented with CORT for 4 weeks prior to mating. Interestingly in this model, the F1 offspring displayed gender segregation between phenotypes, with F2 offspring exhibiting distinct phenotypes compared to that of F1. Similarly, the sperm sncRNA profile in this paternal stress model also changed, displaying elevated levels of three miRNAs that are predicted to target multiple growth factors [166]. Another recent study has explored the potential source of the altered sperm sncRNA profile in chronic stress-exposed mice. Chan et al. have shown that sperm incubated with EVs produced by CORT-treated epididymal epithelial cells, which emulate stress exposure, produce offspring with altered neurodevelopment and stress reactivity during adulthood. Long-lasting changes have been discovered in both protein and miRNA profiles of these EVs, revealing a somatic mechanism of how the paternal environment communicates with germ cells during intergenerational transmission [151].

A more recent study has suggested that sperm miRNA, and potentially rsRNAs, also mediates depression susceptibility of offspring in an intergenerational but not transgenerational manner. F1, but not F2, offspring of the male depression-like model developed depressive-like behaviors when exposed to slight chronic variable stress lasting for 2 weeks. Both the depressive F0 and F1 showed aberrant neuronal gene activation, enhanced HPA axis activity, and impaired synaptic transmission. Altered expression profiles of miRNAs as well as rsRNAs were detected in sperm of the F0 depression-like model. Again, microinjection of the small RNAs (<200 nt) from those sperm into normal oocytes reproduced the depressive-like phenotype in offspring. Remarkably, this injection of small RNAs induced dysregulation of several genes with known neurological functions detected in early embryos. These dysregulated genes were also predicted to be direct targets of differentially expressed miRNAs in sperm of the F0 depression-like model, conveying the possible existence of an altered transcriptional cascade induced by sperm-borne sncRNAs [169].

### 6.4. Paternally Acquired Cognitive Benefit May Also Be Transmitted to Offspring via Sperm-Borne sncRNAs

Although the majority of studies have focused on uncovering inheritance patterns of adverse phenotypes via sperm-borne sncRNAs, a few studies have also shown that paternally acquired cognitive benefit may also be transmitted to offspring. A mouse model of voluntary wheel running was used to investigate the transgenerational effect of paternal exercise. The male, but not female, offspring of the runners exhibited anxiolytic behaviors. Surprisingly, only a few miRNAs and tsRNAs showed alteration in the sperm of runners [188]. In humans, endurance training in healthy individuals was found to affect the expression of eight piRNAs in sperm [189]. Another study exposed adult male mice to an environmental enrichment (EE) paradigm for 10 weeks, which mimicked a combination of physical exercise and cognitive training. This long duration of EE exposure led to enhanced hippocampal synaptic plasticity and memory function, which is transmittable in an intergenerational manner. Microinjection of RNA from sperm of EE mice into normal zygotes also reproduced this cognitive benefit in the resulting offspring. Two miRNAs, miR-132 and miR-212, were upregulated both in the sperm and hippocampus of mice with 10 weeks of EE exposure [170].

## 7. sncRNAs in Sperm, Seminal Plasma, Follicular Fluid, and Embryo Culture Medium as Biomarkers for Fertility and Embryo Development Potential

sncRNAs in biological fluid hold great potential for use as novel biomarkers in various diseases, such as ovarian cancer [206,207], and are therefore of great interest to the development of liquid biopsy. Liquid biopsy is a promising diagnostic surrogate for conventional solid biopsy in the clinic as it is less or non-invasive compared to solid biopsy, yet still provides equally comprehensive and representative information reflecting the state of the tissue. In biological fluid, extracellular sncRNAs are normally encapsulated in exosomes or bound with carrier proteins [207,208,209]. These associations enhance the stability of extracellular sncRNAs, allowing for their detection and use as biomarkers. Previous studies have profiled the sncRNA population in spermatozoa, seminal plasma, follicular fluid, and embryo culture medium, with an emphasis on identifying specific sets of sncRNAs whose relative abundance show strong correlation with reproductive diseases, infertility, or embryo developmental potential [119,210,211,212,213].

Given the abundance of spermatozoa and existence of noninvasive retrieval methods, sperm analysis is standard during fertility evaluation. It is widely agreed upon that the miRNA profile of mature spermatozoa can be used as biomarkers for impaired spermatogenesis [119,210]. Differential expression of several miRNAs was detectable in asthenozoospermic and oligoasthenozoospermic males and compared to normozoospermic males [122]. Another study revealed an aberrant miRNA profile in oligozoospermia males [214]. The miRNA profile of a sperm cell may also impact early embryonic quality. High hsa-mir-191-5p level in human sperm is associated with better early embryo quality, and is therefore considered a potential marker to screen high-quality sperm for enhancing success rates of in vitro fertilization (IVF) [215]. A recent study further indicates that the sncRNA profile of normal sperm selected by classical semen parameters can still be divergent. A panel of miRNA, tsRNA, and rsRNA in sperm has been identified as being significantly associated with embryo quality and they are considered viable biomarkers for screening sperm for IVF [23]. Furthermore, expression profiles of sperm sncRNAs are altered following exposure to environmental stress and toxicants, including cigarettes [185,216], alcohol [183,184], endocrine disruptors [186,187], and air pollution [181]. Alterations in sperm RNA code due to environmental exposure may not affect sperm morphology, but will nonetheless transmit adverse information to subsequent generations. Overall, these studies reinforce the necessity of involving additional epigenetic biomarkers such as sncRNAs for assessing sperm quality.

Seminal plasma, which is partly derived from epididymal fluid, generates sncRNA profiles that implicate seminal plasma quality in and male fertility. miRNA profiles in seminal plasma are correlated with specific spermatogenic defects and, more broadly, in male infertility [217,218,219]. Effects of environmental exposures on spermatogenesis, such as heat stress, may also be reflected by the miRNA profile of seminal plasma [220]. tsRNAs were found to be abundant in human semen-derived exosomes [221]. A recent study focusing on the tsRNA profile in seminal plasma has identified several 5′ tRFs that are elevated in individuals with repeated failure in ICSI [222]. A panel of piRNAs displays differences between fertile and infertile males and five of those piRNAs may potentially distinguish asthenozoospermic and azoospermic from normozoospermic individuals [223].

Components of follicular fluid, including sncRNAs, are likely critical determinants of oocyte quality [211]. Multiple miRNAs in follicular fluid have been reported for their correlations with reproductive diseases such as polycystic ovarian syndrome [224,225,226,227] and premature ovarian dysfunction [228]. Interestingly, miRNAs, specifically miR-320, in human and mouse follicular fluid appear to be another determinant of embryo quality [229], although the underlying mechanism of this correlation remains unknown. Embryos cultured in vitro also produce extracellular vesicles that contain sncRNAs [230]. With constantly improving technologies, various miRNAs, piRNAs, and tRNA halves (tiRNAs) are detectable in spent culture media and suggest indicative potential for embryo quality, such as ploidy and pregnancy outcomes [212,213,230,231,232,233,234,235].

The major challenge of using sncRNA profiles from the aforementioned biological fluids for clinical diagnosis is that sncRNA profiling can be extensively affected by technical differences in materials (e.g., culture media and supplements) [230,233], sample handling procedures (e.g., contaminations and degradation), and detection methods (e.g., qRT-PCR, microarray, or NGS) [236]. Furthermore, a recent study, using both microarray and NGS, indicated that miRNAs were either not altered or not reliably detected in the spent culture medium of a single human blastocyst. This suggests miRNAs in spent culture medium may not be suitable as biomarkers for embryo quality [235]. In general, sncRNA signatures currently reported by different studies tend to be divergent, thus necessitating thorough cross-validation to determine their clinical utility. Culturing techniques, handling, and detection procedures to yield these signatures will also need to be standardized. If cross-validated, those sncRNA signatures in biological fluid can be valuable biomarkers beyond morphological criteria for sperm, oocyte, and embryo selection during the IVF cycle. As mentioned in the previous section, the sncRNA profiles of mature spermatozoa reflect paternal exposures. It is possible that the sncRNA profiles in seminal plasma and follicular fluid also respond to parental exposures and are, therefore, potentially indicative of the effect of certain exposures on fertility and offspring wellbeing.

## 8. Conclusions and Future Perspectives

Across the past few decades, infertility has become a prevailing impairment to human health, affecting nearly one in six couples worldwide [141,142]. A significant portion of infertility cases are idiopathic and fertility continues to decline globally [141]. It is of utmost importance to identify the pathologies of idiopathic infertility and the molecular underpinnings of deteriorating fertility, as well as to improve the assisted reproduction technology (ART). Multiple risk factors for infertility or subfertility have been reported, including unhealthy lifestyles and exposure to toxicants [142,237,238,239]. Recent studies have further indicated that some of those risk factors have intergenerational and even transgenerational effects, emphasizing the urgency to understand the true hereditary mechanisms of infertility [157,158,159].

In this review, we have discussed the relevance of sncRNAs in the pathology of infertility. As biomedical technology continuously advances, novel species of sncRNAs, as well as novel functions and regulation of known sncRNAs, have been uncovered during mammalian germ cell development. Those novel findings imply the existence of a highly complex and dynamic network of sncRNAs in the mammalian germline, where the functions and regulations of sncRNAs correlate to RNA modifications [156], RNA binding proteins [52], interaction with other ncRNAs [132], and potentially RNA structures [11]. Knowledge about this network will offer new perspectives for the pathogenesis and treatment of idiopathic infertility.

The roles of paternal sncRNAs in epigenetic inheritance have been relatively well-established, yet far less is known about how the environmental exposures are translated into the sperm RNA code, as well as how this sncRNA-encoded signal is maintained and amplified into the phenotypes observed in adults. A few studies have suggested the involvement of ROS and oxidative stress in sncRNA regulation in sperm, but detailed mechanisms remain to be explored [204,205,240]. Another study has demonstrated that circulating factors such as metabolites may be altered in response to paternal experience and convey information to the germline [241]. With regard to the functions of sperm-borne sncRNAs in early embryos, it is speculated that a “butterfly effect” occurs [169]. In this “butterfly effect” scenario, the paternally inherited sncRNAs in early embryos directly affect the transcriptional network and/or translation activity. In the meantime, this RNA code may somehow be translated into more stable epigenetic modifications. Those events form a cascade that amplifies the paternally inherited signal and, in the end, affects the whole organism in adulthood [155]. Still, evidence that unequivocally links sncRNA, transcriptional or epigenetic alteration in the embryo, and the acquired phenotypes is yet to exist. It is worth noting that maternal sncRNAs stored in oocytes are likely to be equally, if not more, important than sperm-borne ncRNAs [190]. However, due to confounding factors such as in utero variations, much less is known about the effect of altered expression of maternal ncRNAs.

Finally, we have highlighted the possibility of using sncRNAs in biological fluid as biomarkers for diagnosis of fertility as well as improvement of ART. Although the idea of sncRNAs as biomarkers for fertility and embryo quality is inspiring and promising, extensive work remains to be undertaken in validating their clinical utilization, standardizing clinical procedures, and improving the accuracy and sensitivity of their detection. For example, as RNA modifications have been shown to abort NGS, removal of RNA modifications must be employed when NGS is used as the detection method. Additionally, as bioinformatics analysis is vital to NGS data interpretation, a reliable pipeline is required for procedural standardization [6]. Databases curating reproduction-related sncRNAs, as demonstrated by the SpermBase [242] and Mammalian ncRNA-Disease Repository (MNDR) [243], will also be highly beneficial for the utilization of sncRNAs as fertility biomarkers. Overall, sncRNAs are critical for mammalian reproduction. Various classes of sncRNAs participate in the development of the mammalian germline, with some mediating the transmission of paternal life experience. The family of discovered sncRNAs has been expanding in recent years, with the biogenesis and functions of the newest members being largely unelucidated. Novel functions and regulations were also reported for the relatively well-studied miRNAs, endo-siRNAs, and piRNAs. Further discoveries within this complex realm of sncRNAs will unequivocally benefit the diagnosis and treatment of infertility, as well as understanding of DOHaD.

## Figures and Tables

**Table 1:** Summary information of environmental exposure, altered sperm sncRNA profiles, and the influence on offspring. “ND” indicates transgenerational effects are not evaluated.

Species	Environmental Exposures/Paternal Phenotype	Offspring Phenotype	Intergenerational or Transgenerational Phenotype	Epigenetic Alteration in Sperm	Reference
Psychological effects					
Mouse	Chronic variable stress	Reduced HPA stress axis responsivity	ND	Upregulation of nine miRNAs	[31,165]
Mouse	Elevated paternal glucocorticoid exposure	Various behavioral changes, including hyperanxiety-like and depressive-like behavior	Transgenerational	Elevated levels of three microRNAs, miR-98, miR-144, and miR-190b	[166]
Mouse	Early traumatic stress (MSUS)	Multiple behavioral and metabolic changes, including food intake, insulin hypersensitivity, increased body weight, increased risk-taking, and behavioral despair	Transgenerational	Alteration in multiple sncRNAs and lncRNAs	[167,168]
Mouse	Depression-like model (chronic mild stress induced)	Increased susceptibility to depression	Intergenerational	Total of 19 miRNAs, 24 piRNAs, and 45 rsRNAs show altered expressions	[169]
Mouse	Environmental enrichment paradigm	Enhancement of synaptic plasticity and cognition	Intergenerational	Upregulation of miR 212/132	[170]
Metabolic effects					
Mouse	High-fat diet	Obesity; metabolic disorders in the F1 offspring including glucose intolerance and insulin resistance; altered gene expression of metabolic pathways in early embryos and islets of F1 offspring	ND	Altered expression and RNA modification of tsRNAs, mainly 5′ tRNA halves	[29]
Mouse	Low-protein diet	Altered hepatic cholesterol biosynthesis	ND	Decreased miRNA let-7 levels; increased level of 5′ tRFs derived from glycine tRNAs	[30,171]
Mouse	Western-like diet	Obesity and metabolic pathologies including insulin resistance	Transgenerational	mir-19b	[172]
Mouse	Multigenerational exposure to Western-like diet (WD)	Offspring of the fifth generation of WD-fed male are overweight but with normal glucose metabolism and without fatty liver	Transgenerational	sncRNA signature in the first generation of WD-fed male, such as Increase in rsRNAs, tends to disappear in the fifth generation of WD-fed male	[173]
Mouse	Maternal overnutrition transmitted via paternal lineage	Hedonic behaviors and metabolic defects with gender segregation in F3, i.e., F3 males exhibiting metabolic defects, while females exhibiting addictive-like behaviors	Transgenerational	Elevated level of tsRNAs, predominantly 5′ tRNA halves	[174,175]
Rat	High-protein diet	Increased insulin sensitivity in male but not female F1	ND	Decrease in miRNAs; increase in tsRNAs; increase of a 42 nt mitochondrial rsRNA	[176]
Human	Obesity and bariatric surgery	/	ND	Altered miRNA content in sperm	[177]
Human	Obesity	/	ND	Decreased miR-21 and miR-155; increased miR-122	[178]
Human	6-week diet intervention enriched in vitamin D and omega-3 fatty acids	/	ND	Alteration of 3 tRFs, 15 miRNAs, and 112 piRNAs	[179]
Human	Heathly diet followed by high-sugar diet in two weeks	/	ND	Increase of nitRNA and mitochondrial tsRNA and rsRNA	[180]
Others effects					
Mouse	PM2.5	Male-specific hypophagia, weight loss in general accompanied with decreased liver and kidney massed but increased adipose mass increase	Transgenerational	mmu-mir6909-5p	[181]
Mouse	Alcohol	Diverse metabolic and behavioral changes in adulthood; late-term fetal growth restriction and a loss of placental efficiency	ND	Altered expression of multiple sncRNAs; reduced epididymal expression of a tRNA methyltransferase, Nsun2	[182,183,184]
Mouse	Cigarette	Mild increase in body weight of F1	ND	Altered miRNA profile	[185]
Rat	Maternal (gestation) exposure to endocrine disruptors transmitted via paternal lineages	Increased incidence of male infertility in vinclozolin-exposure studies; increased susceptibility to obesity in DDT-exposure studies; susceptibility to diseases in multiple organs and immune system in both cases	Transgenerational	Concurrent alterations in DNA methylation, ncRNA, and histone retention	[186,187]
Mouse	Exercise	Male-specific anxiolytic behaviors	ND	Increase in miR-19b; decrease in miR-455 and miR-133a; increased tRNA-Gly- and decreased tRNA-Pro-derived RNAs	[188]
Human	Endurance training	/	ND	Altered expression of 8 piRNAs	[189]

## Data Availability

Not applicable.
